# Physiologically Relevant Oxygen Concentration (6% O_2_) as an Important Component of the Microenvironment Impacting Melanoma Phenotype and Melanoma Response to Targeted Therapeutics In Vitro

**DOI:** 10.3390/ijms20174203

**Published:** 2019-08-27

**Authors:** Marta Osrodek, Mariusz L. Hartman, Malgorzata Czyz

**Affiliations:** Department of Molecular Biology of Cancer, Medical University of Lodz, 6/8 Mazowiecka Street, 92-215 Lodz, Poland

**Keywords:** melanoma, normoxia, hypoxia, MITF, PGC1α, cellular metabolism, cancer heterogeneity, vemurafenib, trametinib

## Abstract

Cancer cell phenotype largely depends on oxygen availability. The atmospheric oxygen concentration (21%) used in in vitro studies is much higher than in any human tissue. Using well-characterized patient-derived melanoma cell lines, we compared: (i) activities of several signaling pathways, and (ii) the effects of vemurafenib and trametinib in hyperoxia (21% O_2_), normoxia (6% O_2_) and hypoxia (1% O_2_). A high plasticity of melanoma cells in response to changes in oxygen supplementation and drug treatment was observed, and the transcriptional reprograming and phenotypic changes varied between cell lines. Normoxia enhanced the expression of vascular endothelial growth factor (VEGF), glucose metabolism/transport-related genes, and changed percentages of NGFR- and MITF-positive cells in cell line-dependent manner. Increased protein stability might be responsible for high PGC1α level in MITF^low^ melanoma cells. Vemurafenib and trametinib while targeting the activity of MAPK/ERK pathway irrespective of oxygen concentration, were less effective in normoxia than hyperoxia in reducing levels of VEGF, PGC1α, SLC7A11 and Ki-67-positive cells in cell line-dependent manner. In conclusion, in vitro studies performed in atmospheric oxygen concentration provide different information on melanoma cell phenotype and response to drugs than performed in normoxia, which might partially explain the discrepancies between results obtained in vitro and in clinical settings.

## 1. Introduction

As our understanding of the role of tumor microenvironment (TME) in cancer biology and disease progression has expanded, numerous attempts to mimic TME in in vitro settings have been made. These include three-dimensional matrices [1,2], co-cultures with other cell types [3,4] and modifications of culture media supplementation [5,6,7,8]. However, in spite of oxygen being an essential biological component, its concentration characteristic for human tissues is misrepresented in standard in vitro research models and majority of studies in vitro are performed in an atmospheric oxygen concentration (21% O_2_) [9]. Mammalian cells are exposed to up to 14.5% oxygen in vivo, and distinct cell functions are attributed to tissue-specific oxygen levels [10]. Furthermore, there is accumulating evidence of the detrimental effect of the atmospheric oxygen level in cell culture, particularly affecting primary, non-transformed cells and stem cells [11,12,13,14]. Although cancer cells are more resilient and are easily propagated ex vivo, oxygen remains a crucial, regulatory factor in multiple aspects of cancer biology [9]. It is therefore not only intuitive but rational that ex vivo studies on mammalian cells should be conducted at oxygen concentrations that are more appropriate for the tissue they originated from.

Melanoma arises from malignant transformation of melanocytes residing in the epidermal layer of skin, where the oxygen concentration does not exceed 8% O_2_ [9,15]. Most melanomas harbor activating mutations in genes associated with the RAS/RAF/MEK/ERK pathway, with BRAF^V600E^ being the most prevalent [16]. Efforts to target this pathway led to the development of selective therapeutics against BRAF^V600E^ or MEK1/2 kinase, however, the vast majority of melanomas are either intrinsically resistant or develop drug resistance within the first year of treatment [17,18,19,20]. Despite being rather small in size, melanoma tumors, like other solid cancers, comprise poorly oxygenated areas of hypoxia, which prompted multiple studies focused on the role of hypoxic conditions in modifying melanoma phenotype and melanoma response to treatment [21,22,23,24]. Little is known, however, how higher but still physiological oxygen concentrations affect melanoma cell phenotype and the predominant research approach in all oxygen-related studies is a short-term exposure of melanoma cells to low (≤1%) oxygen concentrations following standard hyper-oxygenated cell culture.

In recent years, metabolic reprogramming has gained significant interest. In spite of previous reports showing that cancer cells are primarily glycolytic, it has become clearer that melanomas manifest great metabolic plasticity and heterogeneity. While enhanced glycolysis favors metastatic behavior in melanoma cells and several elements of the glycolytic pathway are considered as prognostic markers [25,26], some melanoma cells characterized by high expression of the peroxisome proliferator-activated receptor γ coactivator 1α (PGC1α), exhibit greater dependency on oxidative phosphorylation [27]. Moreover, results showing contradictory metabolic phenotypes have been obtained between in vitro and in vivo studies. For instance, while ovarian tumor-initiating cells are highly glycolytic in vivo they display a strong oxidative metabolism in vitro [28].

Three melanoma cell lines derived from surgical specimens of previously untreated patients, DMBC12, DMBC17, and DMBC28, were used in this study. They are genetically and phenotypically different, as published in detail elsewhere [29,30,31]. Briefly, two of the cell lines, DMBC12 and DMBC28, harbor V600E substitution in BRAF and one, DMBC17, harbors Q61R substitution in HRAS. As the microphthalmia-associated transcription factor (MITF) is one of the major regulators of mitochondrial respiration in the melanocytic lineage [27], we have chosen melanoma cell lines that differ in the expression and activity of MITF [31], with the highest in DMBC17 cells and almost undetectable in DMBC12 cells. When a multi-stage differentiation model [32] is applied to classify melanoma cells chosen for this study, and the expression of the sex determining region Y-box 10 (SOX10) and tyrosine-protein kinase receptor (AXL) is considered in addition, each cell line represents a different phenotype: DMBC12 cell line (MITF^low/no^/SOX10^high^/AXL^high^) can be considered as invasive/neural crest-like, DMBC28 cell line (MITF^high^/SOX10^high^/AXL^low^) as neural crest/pigmentated, and DMBC17 cell line (MITF^high^/SOX10^high^/AXL^low^) as melanocyte-like [31].

We established an in vitro culture model of patient-derived melanoma cells in a physiological oxygen concentration corresponding to human peripheral tissues (6% O_2_). These conditions were defined in this study as normoxia, whereas standard laboratory conditions (21% O_2_) were designated as hyperoxia. We aimed to compare the phenotypes of investigated melanoma cell lines in different concentrations of oxygen. The influence of two drugs currently used in melanoma treatment, vemurafenib, a BRAF^V600E^ inhibitor and trametinib, a MEK1/2 inhibitor, was also compared in these conditions.

## 2. Results

### 2.1. In Vitro Culture of Melanoma Cells in 6% O_2_ Alters their Metabolic Demands

**Figure 1:**
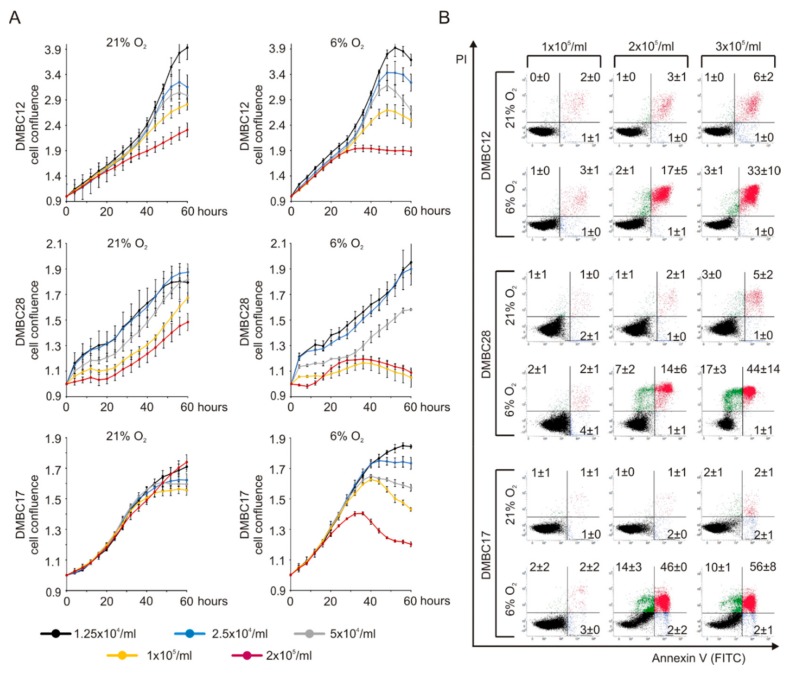
Proliferation and survival of melanoma cells cultured in 6% O_2_ greatly depend on melanoma cell density. (**A**) Proliferation time-courses of melanoma cells of varying seeding densities in 21% O_2_ and 6% O_2_. Cell proliferation was measured as changes in the occupied area over time using IncuCyte, and shown as fold-change relative to the confluence at time T_0_. (**B**) Representative dot plots of Annexin V/propidium iodine (PI) stained cells after 72 h in culture, showing early apoptotic (blue), late apoptotic (red), and necrotic (green) cells. Numbers in each quadrant are percentages of cells (mean values of three biological replicates ± SD). DMBC, melanoma cell populations obtained at the Department of Molecular Biology of Cancer from surgical specimens.

First, we investigated proliferation of melanoma cells and observed that initial changes in cell confluence were similar in hyperoxia and normoxia, however, after approximately 30 h a strong cell density-dependent inhibition was detected in normoxia (Figure 1A). Moreover, there was a pronounced reduction of cell survival after three days in 6% O_2_, and Annexin V/propidium iodide staining revealed that apoptotic and necrotic cells could be counted, especially in DMBC17 and DMBC28 cell line cultures (Figure 1B). While the percentages of apoptotic and necrotic cells were low in hyperoxia, even at initially high density of cells reaching 3 × 10^5^/mL, in normoxia both apoptosis and necrosis were induced already at lower seeding density of 2 × 10^5^ cells/mL (Figure 1B). This might indicate that melanoma cells differ in metabolic demands when cultured in the presence of 6% and 21% of oxygen.

### 2.2. Oxygen Concentration-Dependent Changes in the Activity of Signaling Pathways are Diverse among Melanoma Cell Lines

**Figure 2:**
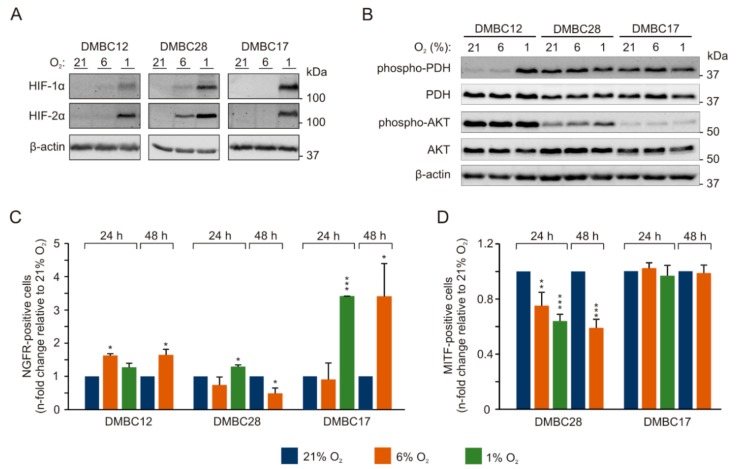
Phenotypic alterations in melanoma cells in different oxygen concentrations depend on initial phenotype of melanoma cells. Melanoma cells were incubated at different oxygen concentrations, 21% O_2_ (hyperoxia) 6% O_2_ (normoxia) and 1% O_2_ (hypoxia). Protein levels of (**A**) HIF (hypoxia-inducible factor)-1α and HIF-2α and (**B**) p-PDH (phosphorylated pyruvate dehydrogenase, inactive form), PDH (total), p-AKT (phosphorylated protein kinase B) and total AKT were assessed by immunoblotting with representative images shown. β-actin was used as a loading control. The percentages of (**C**) NGFR (nerve growth factor receptor)-positive cells and (**D**) MITF (microphthalmia-associated transcription factor)-positive cells in normoxia and hypoxia relative to the standard culture conditions (21% O_2_). MITF-positive cells were almost undetectable in DMBC12 cell population (see Figure S1). *n* = 3, except for hypoxia (*n* = 2). Differences are considered significant at * *p* < 0.05, ** *p* < 0.01, *** *p* < 0.001.

Oxygen plays a fundamental role in tumor biology, and several signaling molecules respond to oxygen deficit. In three different oxygen concentrations, we assessed closely interconnected hypoxia-inducible factor (HIF)-1α and HIF-2α as the key regulators of the cellular response to oxygen deprivation, phosphorylation status of the pyruvate dehydrogenase (PDH) crucial for conversion of pyruvate into acetyl coenzyme A (acetyl-CoA), and the activity of the PI3K/AKT (phosphoinositide 3- kinase/protein kinase B) pathway promoting HIF-1α activity. HIF-1α and HIF-2α, whose protein levels were markedly increased in hypoxia in all cell lines, were also clearly detectable already in normoxia in DMBC28 cells (Figure 2A). While phosphorylation of PDH was constant in DMBC17 and DMBC28 cells, irrespective of oxygen concentration, DMBC12 cells exerted high PDH phosphorylation only in hypoxia, which indicates that PDH was active only in DMBC12 cells in normoxia and hyperoxia (Figure 2B). Activity of the PI3K/AKT pathway varied between cell lines in hyperoxia and was differently altered when cells were exposed to lower oxygen concentrations (Figure 2B). While activity of AKT was gradually increased in normoxia and hypoxia in phospho-AKT^low^ DMBC28 cells, in another phospho-AKT^low^ DMBC17 cell line, no increase was observed at lower oxygen concentrations (Figure 2B). In DMBC12 cells, a high level of phospho-AKT was detected in all oxygen conditions.

### 2.3. Oxygen Concentration-Dependent Changes in the Composition of Melanoma Cell Populations

In the following experiments, the percentages of nerve growth factor receptor (NGFR)- and MITF-positive cells were compared between cell populations grown in different oxygen concentrations (Figure 2C,D). In DMBC12 cell population, NGFR was expressed by 15.2 ± 1.5% cells in hyperoxia, and this percentage was significantly but only slightly higher in normoxia. In NGFR^low^ DMBC17 cell population (1.9 ± 0.4% in hyperoxia) it was significantly higher in both normoxia after 48 h and hypoxia already after 24 h. DMBC28 cell line, with 20.6 ± 4.3% NGFR-positive cells in hyperoxia, was exceptional as lowering concentration of oxygen to 6% significantly reduced the percentages of NGFR-positive cells after 48 h. Percentages of MITF-positive cells in MITF^high^ cell lines were either significantly lower in normoxia and hypoxia than in hyperoxia (DMBC28) or remained unchanged (DMBC17). This suggests that melanoma cells cultured in vitro in the presence of 21% O_2_ may differ in their phenotypes from melanoma cells grown in vivo at much lower oxygen concentrations.

### 2.4. Normoxia Promotes the Expression of Glucose Metabolism/Transport-Related Genes and to the Lower Extent Genes Associated with Glutamine Metabolism and Transport

**Figure 3:**
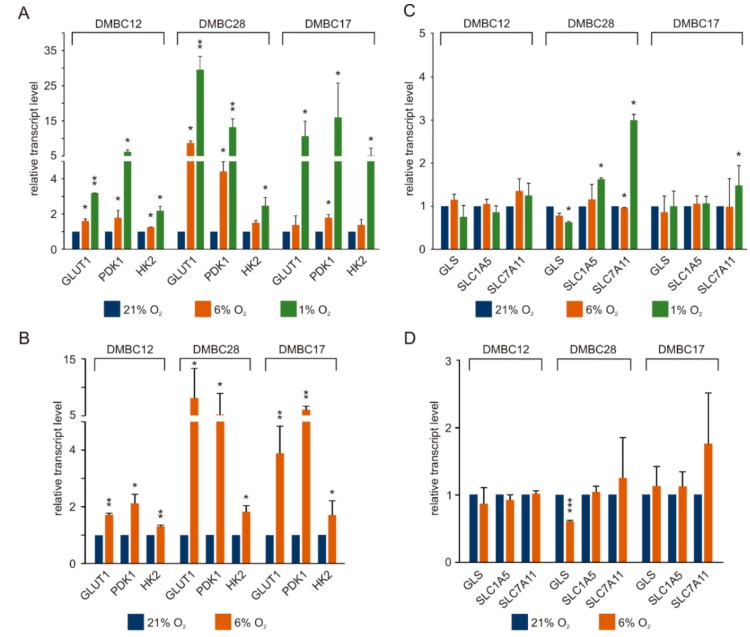
Normoxia stimulates the expression of genes associated with glucose metabolism and to the lower extent with glutamine metabolism in cell line-dependent manner. (**A**) Transcript levels of GLUT1 (glucose transporter 1), PDK1 (pyruvate dehydrogenase kinase 1) and HK2 (hexokinase 2) in melanoma cells incubated in the presence of 21% O_2_, 6% O_2_ or 1% O_2_ for 24 h were determined by qRT-PCR and normalized to the expression of a reference gene RPS17. Gene expression in 6% O_2_ and 1% O_2_ is presented relative to the expression in 21% O_2_. (**B**) Transcript levels of GLUT1, PDK1 and HK2 in melanoma cells cultured in the presence of 6% O_2_ for at least 3 weeks (established 6% O_2_ culture) relative to their levels in cells cultured in 21% O_2_. (**C**) Transcript levels of GLS (glutaminase), SLC1A5 (solute carrier family 1 member 5) and SLC7A11 (solute carrier family 7 member 11 transporter) in melanoma cells after 24 h incubation in 21% O_2_, 6% O_2_ and 1% O_2_, or (**D**) in the established 6% O_2_ culture, relative to their levels in 21% O_2_. Bars represent mean values of 3-4 biological replicates ± SD. Differences are considered significant at * *p* < 0.05, ** *p* < 0.01 or *** *p* < 0.001.

The expression of pivotal glucose and glutamine metabolism/transport-related genes was assessed in melanoma cells exposed to 6% O_2_ and 1% O_2_. As the reference, the expression of these genes in 21% O_2_ was used. We analyzed the expression of genes encoding glucose transporter 1 (GLUT1), hexokinase 2 (HK2), the first enzyme of the glycolytic pathway, and pyruvate dehydrogenase kinase 1 (PDK1), a metabolic gatekeeper, which inhibits the activity of PDH and restrains pyruvate entry to the TCA cycle. All these genes are direct targets of HIF-1α. Accordingly, the expression of all three genes was significantly enhanced when cells were exposed to hypoxia for 24 h (Figure 3A).

PDK1 transcript levels were significantly increased also in normoxia, and this enhancement was especially high in DMBC28 cells. Normoxia induced a significant increase of GLUT1 mRNA levels in DMBC12 cells and DMBC28 cells, whereas the expression of HK2 was significantly increased only in DMBC12 cells. These results show that investigated melanoma cell lines vary in their reaction to a transition from hyperoxia to normoxia. We hypothesized that metabolic adaptation might be required, and indeed when melanoma cells were cultured in normoxic conditions for 3 weeks, the transcript levels of GLUT1, PDK1 and HK2 were markedly enhanced also in DMBC17 cells (Figure 3B). Although significantly changed, the expression of HK2 was the least responsive to hypoxia in all cell lines and proportionally, changes in HK2 expression were the least pronounced also in normoxia. In the following experiments, melanoma cells cultured in normoxia for at least 3 weeks were used.

First however, we investigated whether the expression of genes involved in glutamine uptake and metabolism is subjected to oxygen-dependent regulation after short and long exposure to low oxygen levels (Figure 3C,D). We assessed the transcript levels of solute carrier family 1 member 5 (SLC1A5, also known as ASCT2), which is the main transporter of glutamine into the cell, glutaminase (GLS), which catalyzes the first step of glutamine metabolism by converting glutamine to glutamate, and solute carrier family 7 member 11 transporter (SLC7A11), which exports glutamate in exchange for cystine. When the expression of glutamine-related genes was compared in normoxic and hyperoxic conditions, no significant differences were observed with the exception of SLC7A11 transcript in DMBC28 cells shortly exposed to 6% O_2_ (Figure 3C), and GLS transcript in DMBC28 cells exposed to normoxia for 3 weeks (Figure 3D).

### 2.5. Increased Protein Stability Might be Responsible for High PGC1α Level in MITF^low^ Melanoma Cells

**Figure 4:**
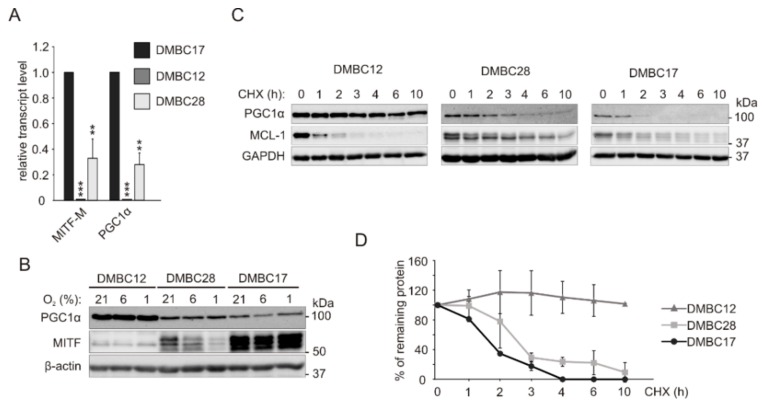
PGC1α (peroxisome proliferator-activated receptor γ coactivator 1 alpha) protein accumulates in MITF^low^ melanoma cells. (**A**) Transcript levels of MITF-M and PGC1α were determined by qRT-PCR and normalized to the expression of a reference gene RPS17. Gene expression in DMBC12 and DMBC28 cells is shown relative to the expression in DMBC17 cells. Bars represent mean values of 3 biological replicates ± SD. Differences are considered significant at ** *p* < 0.01, *** *p* < 0.001. (**B**) Protein levels of MITF and PGC1α in different oxygen concentrations were assessed by western blot with representative images shown (*n* = 3). β-actin was used as a loading control. (**C**) For protein decay assay, melanoma cells were treated with 100 μg/mL cycloheximide (CHX) and protein levels of PGC1α and MCL-1 (myeloid cell leukemia 1) were determined at indicated time points. MCL-1, having short half-life, served as control of protein stability. GAPDH was used as a loading control. (**D**) Quantification of average protein levels of PGC1α calculated from two independent experiments.

Following the intriguing results of the analysis of PDH phosphorylation, which suggest that only DMBC12 cells might rely on mitochondrial metabolism in normoxic and hyperoxic conditions, we investigated the influence of oxygen level on the expression of PGC1α responsible for immediate cell adaptation for high energetic demands, and MITF, one of the key regulators of PGC1α expression in melanoma cells. Analysis of transcript levels of MITF and PGC1α showed a good correlation between them, as relative to DMBC17 cell line with the highest levels of MITF and PGC1α mRNAs, both transcripts were at markedly lower levels in DMBC28 cells and were almost undetectable in DMBC12 cells (Figure 4A).

Surprisingly, although MITF protein was almost undetectable in DMBC12 cells, a high level of PGC1α protein was found in these cells (Figure 4B). This altogether indicates that while there is a good correlation between transcript levels of MITF and PGC1α in melanoma cells, no correlation could be found at the protein level. To address this discrepancy, we carried out a protein decay assay. We found that while PGC1α half-life did not exceed three hours in DMBC17 and DMBC28 cells, PGC1α protein remained exceptionally stable in DMBC12 cells (Figure 4C,D).

### 2.6. The Response to Targeted Therapeutics Depends on Oxygen Availability and Original Phenotype of Melanoma Cells

**Figure 5:**
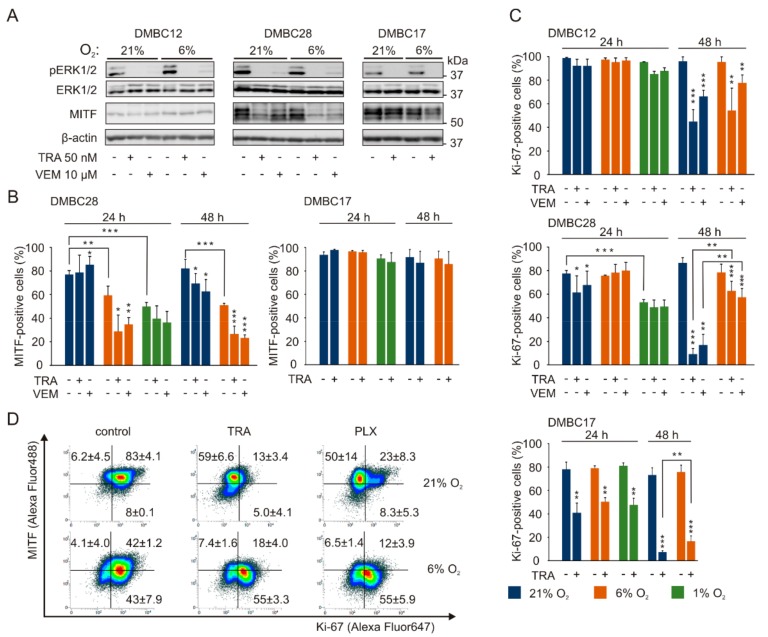
Vemurafenib and trametinib while targeting the MAPK/ERK pathway irrespective of oxygen concentration, affect MITF-positive and Ki-67-positive melanoma cells in oxygen- and cell line-dependent manner. (**A**) The effects of trametinib (TRA) and vemurafenib (VEM) on ERK1/2 activity and MITF level in 21% and 6% O_2_ were determined by Western blotting with representative images shown (*n* = 3). β-actin was used as a loading control. (**B**) The percentage of MITF-positive cells in control cultures and after treatment with either 50 nM trametinib (TRA) or 10 µM vemurafenib (VEM) are shown as mean values ± SD (*n* = 3). Results for DMBC12 were not quantified due to almost undetectable MITF level in control and lack of changes in drug-treated cells. Differences are considered significant at * *p* < 0.05 ** *p* < 0.01 *** *p* < 0.001. See Figure S1 for representative dot plots. (**C**) The percentage of Ki-67-positive cells in control cultures and after treatment with 50 nM trametinib or 10 µM vemurafenib are shown as mean values of 3 biological replicates ± SD. Differences are considered significant at * *p* < 0.05 ** *p* < 0.01 *** *p* < 0.001. See Figure S1 for representative dot plots. (**D**) Representative density plots of dual-stained DMBC28 cells with anti-MITF and anti-Ki-67 antibodies after 48 h of drug treatment. Average percentages of cells positive for one or both markers are shown (*n* = 2).

Next, we investigated whether melanoma cell response to vemurafenib, an inhibitor of BRAF^V600^, and trametinib, an inhibitor of MEK1/2, depends on oxygen concentration. For that we assessed phosphorylation of ERK1/2, an effector protein of the RAS/RAF/MEK/ERK pathway, and MITF, a transcription factor determining the phenotypic state of melanoma cells. While the activity of ERK1/2 was inhibited by vemurafenib and trametinib to an almost undetectable level in all tested cell lines both in normoxia and hyperoxia, MITF level was reduced more substantially in normoxia than in hyperoxia in both MITF^high^ cell lines, DMBC17 and DMBC28 (Figure 5A).

Having observed that drug-induced changes in MITF expression in MITF^high^ cells depend on oxygen concentration when measured at the bulk protein level (Figure 5A), we asked the question whether MITF expression is downregulated uniformly in all cells or a subpopulation of MITF-positive cells is reduced. Flow cytometric analysis of MITF expression revealed that although the percentages of MITF-positive cells were not substantially changed by drug treatment in hyperoxia, they were significantly decreased in DMBC28 cell population cultured in normoxia (Figure 5B, Supplementary Materials Figure S1). Of note, the percentages of MITF-positive DMBC28 cells differed significantly even in control populations when cultured in hypoxic, normoxic and hyperoxic conditions (Figure 5B). The percentages of MITF-positive cells in DMBC17 cell line were similar in all oxygen concentrations, and remained unaltered in trametinib-treated cells (Figure 5B). MITF-positive cells were almost undetectable in DMBC12 cell line irrespective of oxygen concentration, also after drug treatment (Figure S1).

The percentages of Ki-67-positive cells were similar in control cells of all cell lines and regardless of oxygen concentration, except for DMBC28 cells grown in hypoxia, which were significantly lower than in hyperoxia (Figure 5C). Vemurafenib and trametinib reduced the frequency of Ki-67-positive cells to a variable extent in investigated cell lines after 48 h of treatment. Interestingly, drugs reduced the percentage of Ki-67-positive cells to significantly lower level in hyperoxia than in normoxia, which might indicate that melanoma cells in normoxia could be less responsive to drug treatment. The majority of DMBC28 cells that survived drug treatment in hyperoxia were MITF-positive and Ki-67-negative, in contrast to drug-treated melanoma cells in normoxia, which were predominantly MITF-negative and Ki-67-positive (Figure 5D).

### 2.7. Drug-Triggered Reduction of PGC1α Level is Accompanied with Induction of Apoptosis

**Figure 6:**
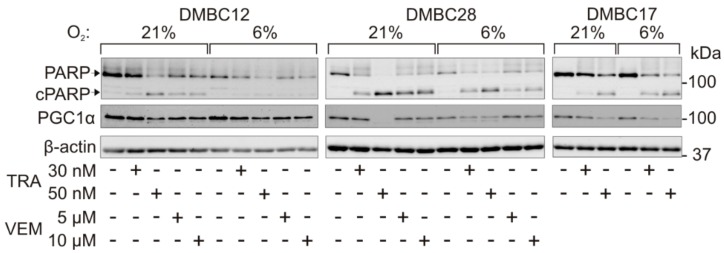
Changes in the protein level of PGC1α during drug-induced apoptosis. Protein levels of PARP and PGC1α after 44 h of treatment with vemurafenib (VEM) and trametinib (TRA) at indicated concentrations in normoxia (6% O_2_) and hyperoxia (21% O_2_) were assessed by western blotting with representative images shown (*n* = 3). Upper and lower arrows indicate full-length and cleaved PARP (cPARP), respectively. β-actin was used as a loading control.

PGC1α has been shown to regulate the survival of PGC-1α-positive melanoma cells, and knockdown of PGC1α significantly induced apoptosis [33]. Our results support and extend these findings as a tendency of drug-triggered PGC1α downregulation in samples with greater PARP cleavage was observed, which might indicate that the stability of this protein can be connected with induction of apoptosis, especially in melanoma cells with lower expression of PGC1α, DMBC28 and DMBC17 (Figure 6).

### 2.8. Vemurafenib and Trametinib Reduce the Expression of VEGF in Hyperoxia but not in Normoxia

**Figure 7:**
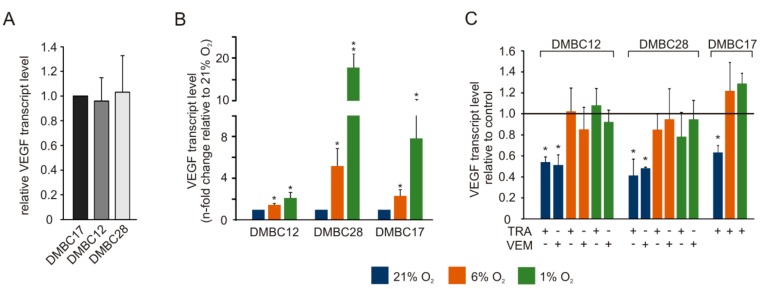
Effects of trametinib (TRA) and vemurafenib (VEM) on VEGF transcript level assessed at different oxygen concentrations. VEGF transcript levels were determined by qRT-PCR and normalized to the expression of a reference gene RPS17. (**A**) Transcript levels of VEGF were determined in cell cultures grown in hyperoxia by qRT-PCR and their expression is compared between cell lines with transcript levels in DMBC17 cells set as one. (**B**) Transcript level of VEGF in 6% O_2_ and 1% O_2_ is presented relative to its level in 21% O_2_. (**C**) Changes in VEGF transcript level after drug treatment is presented relative to its expression in control. Bars represent mean values of 3-4 independent experiments ± SD. Differences are considered significant at * *p* < 0.05, ** *p* < 0.01.

Although melanoma cell lines used in this study did not differ in the basal expression level of *VEGFA* in hyperoxia (Figure 7A), lowering the oxygen level was associated with significant VEGF transcript level enhancement that varied markedly between cell lines with DMBC12 showing the lowest increase (Figure 7B). Interestingly, they were significantly reduced by vemurafenib and trametinib, however, only under hyperoxic conditions (Figure 7C).

### 2.9. Vemurafenib and Trametinib Reduce the Expression of Genes Related to Glutamine and Glucose Metabolism and Transport to a Different Extent and in Cell Line-Dependent Manner

**Figure 8:**
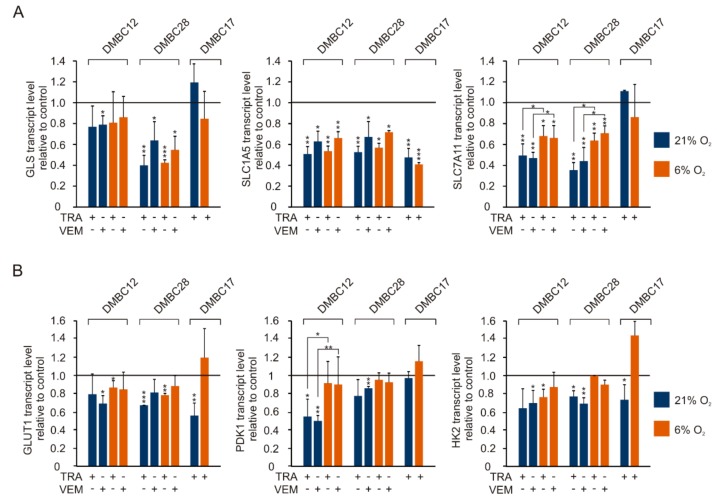
Effects of trametinib (TRA) and vemurafenib (VEM) on the expression of genes related to glutamine and glucose metabolism assessed at different oxygen concentrations. Drug-induced changes in transcript levels of glutamine (**A**) and glucose (**B**) metabolism-related genes after 24 h of treatment with either vemurafenib or trametinib. qRT-PCR data were normalized to the expression of a reference gene RPS17. Bars represent mean values of 3-4 biological replicates ± SD. Statistically significant differences are indicated: * *p* < 0.05, ** *p* < 0.01 or *** *p* < 0.001.

We analyzed changes in the expression of glutamine and glucose metabolism-related genes in response to trametinib and vemurafenib in melanoma cells grown in hyperoxia and normoxia for at least 3 weeks (Figure 8). Transcript levels of SLC1A5 were uniformly and significantly decreased in all melanoma cell lines in normoxia and hyperoxia, which suggests that glutamine uptake is downregulated by vemurafenib and trametinib irrespective of the oxygen availability. SLC7A11 expression was downregulated in both oxygen concentrations in two out of three drug-treated cell lines, whereas GLS expression was markedly and significantly decreased only in DMBC28 cells, which suggests that glutamine export and metabolism might be also downregulated by targeted therapeutics, however, not in all melanomas. The effects of vemurafenib and trametinib on the expression of glucose metabolism-related genes were less pronounced. It is worth to note that effects of drugs on transcript level of PDK1 in DMBC12 cells and SLC7A11 in DMBC12 cells and DMBC28 cells were significantly weaker in normoxia than in hyperoxia.

## 3. Discussion

In vitro cell cultures are an inherent part of cancer research. Much of our knowledge on the molecular mechanisms crucial for cancer maintenance and response to treatment derives from in vitro studies performed in the atmospheric oxygen concentration (21%), which is much higher than in any human tissue. Therefore, despite the undeniable value of cell line-based research, the model is rather flawed and its direct contribution to clinical cancer research is questionable [34]. The main aim of this study was to compare the phenotype of melanoma cells grown in different oxygen concentrations: 21% designated as hyperoxia, 6% chosen as mimicking the physiological oxygen level present in many tissues and regions of tumors and called in this study normoxia, and 1% as hypoxia. In addition, we investigated the oxygen concentration-dependent melanoma cell response to targeted therapeutics, vemurafenib and trametinib.

The patient-derived melanoma cell lines chosen for this study differ in oncogenic mutations in the MAPK/ERK pathway and activity of transcription factor MITF, one of the critical regulators of melanoma phenotype [30,31]. As the oxygen concentration is critical for cancer cell energy metabolism and both, the MAPK/ERK pathway and MITF signaling, have been associated with the control of metabolic activities of melanoma cells [27], the selection of DMBC12 cells (BRAF^V600E^/MITF^low^), DMBC28 cells (BRAF^V600E^/MITF^high^) and DMBC17 (HRAS^Q61R^/MITF^high^) [30,31] for this study was well justified. And indeed, we were able to demonstrate: (i) cell type-dependent phenotypic changes in response to different oxygen concentrations; (ii) oxygen level-dependent effects of targeted therapeutics in melanoma cells.

It has been demonstrated that melanoma development and metastasis are associated with changes in cell metabolism involving a shift from oxidative phosphorylation (OXPHOS) to aerobic glycolysis [35]. The enhanced glycolytic activity was connected to BRAF^V600E^, constitutively active in majority of melanomas [36]. BRAF-dependent metabolic rewiring includes activation of HIF-1α [37], which is normally induced in response to low oxygen levels and leads to increased expression of glucose transporters and glycolytic enzymes. Many melanomas display the activity of HIF-1α also under non-hypoxic conditions [38,39]. In our study, HIF-1α was expressed in BRAF^V600E^ melanoma cells in hypoxia and to a lesser extent in normoxia, whereas in HRAS^Q61R^ melanoma cells oxygen concentration had to be reduced to 1% to stimulate HIF-1α expression. We showed that the expression of glucose transporter/enzyme-related genes was also oxygen-dependent in both BRAF mutant and HRAS mutant melanoma cells, however, while their expression was significantly higher in BRAF mutant DMBC28 cells already after short exposure to normoxia, HRAS mutant DMBC17 cells needed a longer adaptation period to promote the expression of these genes. In addition to maintaining a state of aerobic glycolysis by enhancing the expression of glucose transporters and glycolytic enzymes, HIF-1α participates in controlling PDH enzymatic activity [40]. PDH is a key enzyme of a multienzyme complex catalyzing pyruvate conversion into acetyl-CoA and linking glycolysis to the tricarboxylic acid (TCA) cycle, thus promoting OXPHOS [41]. Under hypoxic conditions, phosphorylation that inactivates PDH follows an upregulation of its kinase, PDK1, which is HIF-1-dependent [42,43]. However, PDH phosphorylation can be also HIF-1-independent, as it can precede the upregulation of PDK1 mediated by HIF-1α [44]. In this study, protein levels of HIF-1α and phosphorylated PDH suggest that DMBC12 cells display an OXPHOS phenotype since similar and low level of HIF-1α could be detected in normoxia and hypoxia, and a strong inactivating PDH phosphorylation occurred in these cells only in hypoxia, whereas DMBC28 and DMBC17 cell lines might be defined as having a glycolytic phenotype with a high level of inactive PDH irrespective of oxygen availability, especially DMBC28 cell line, in which HIF-1α could be detected also in normoxic conditions. An OXPHOS^high^ subset of melanomas shows a high expression rate of PGC1α [33], which is an important transcriptional coactivator regulating oxidative metabolism in many tissues [45,46,47,48,49]. The role of PGC1α is controversial as it can act as a tumor promoter and a tumor suppressor, which can be partially explained by its cell type specificity, including expression and diverse interacting proteins [49,50,51,52]. Multiple promoters of *PPARGC1A*, a gene encoding PGC1α, are selectively responsive in distinct tissues, and MITF was found to directly stimulate the expression of PGC1α by binding to its promoter region in melanoma cells [27]. MITF is a master regulator of melanocytes and a lineage-specific melanoma oncogene [53], and it is important for cell differentiation, proliferation and survival [54,55]. MITF activity is tightly regulated in melanocytes and melanoma [56,57], including transcriptional repression by HIF-1α [58]. A correlation between transcript levels of MITF and PGC1α was previously demonstrated in melanoma cells [27], and this correlation was also confirmed in our study. Importantly, for the first time we have shown that while MITF^low/no^ melanoma cells display a low level of PGC1α transcript, they may accumulate PGC1α protein reaching high level, probably due to mechanism(s) that increase its stability. According to literature, PGC1α protein has an approximate half-life of 2–3 h and multiple factors are responsible for its stability [59]. The mechanism(s) preserving a high level of PGC1α protein in melanoma cells for longer than 10 h needs to be elucidated.

In addition to altering cancer cell metabolism, PGC1α has been implicated in cell survival, and knockdown of PGC1α results in induction of apoptosis in melanoma cells [33]. Our results are in agreement with this notion as we have shown that drug-triggered reduction of PGC1α level is accompanied with induction of apoptosis. Therefore, the paradigm that PGC1α is increased in response to drug-triggered inhibition of ERK1/2 activity [27] might be true unless the inhibition of ERK1/2 is accompanied with induction of apoptosis. Interestingly, in PGC1α^high^ DMBC12 cell line both drug-induced reduction of PGC1α and induction of apoptosis were less pronounced than in PGC1α^low^ cell lines, DMBC28 and DMBC17.

Cell line-specific effects of adjusting oxygen level to this more closely resembling tumor natural one can be also exemplified by variable increase in AKT activity, expression of VEGF and MITF, and cell response to targeted therapeutics in terms of percentages of MITF- and Ki-67-positive cells at the cell population level. Our finding that the activity of the PI3K/AKT pathway is not universally activated in all patient-derived melanoma cell lines exposed to decreased oxygen level, has also been reported in other cancer types [60]. Notably, neither of the cell lines used in our study harbored mutations in PTEN [30], therefore, the mechanism behind high AKT activity in DMBC12 is likely not of genetic background. It has been recently shown that AMPK, a major energy sensor, phosphorylates mTORC2, which leads to phosphorylation of AKT at Ser473 [61]. This could partially explain the increase in phospho-AKT in DMBC28 in hypoxia and high levels of it in the fast proliferating DMBC12 cell line, however, it would not explain lack of AKT activity in DMBC17. Moreover, we observed an overlapping activity of AKT and expression of PGC1α, which is also activated by AMPK [62].

The effect of targeted therapeutics on the expression of glutaminase and glutamine transporters in drug-sensitive, patient-derived melanoma cells have not been described before. However, several reports indicate the role of mitochondrial metabolism of glutamine in drug-resistance [63,64]. In those studies, melanoma cells with acquired resistance to BRAF^V600E^ or MEK inhibitors depended on glutamine metabolism for survival and were susceptible to glutaminase inhibition. The relevance of glutamine transport in melanoma is supported by data indicating that *SLC1A5* and *SLC7A11* are overexpressed in melanoma cells when compared to melanocytes [65,66]. Whether the expression of these genes would be restored in resistant cells remains to be clarified. The selective increase in *SLC7A11* expression in hypoxic DMBC28 cells is unclear, however, such increase has been previously described in breast cancer [67] and renal cancer cell lines [68], due to high HIF activity or glucose starvation, respectively.

The MITF/PGC1a axis has also been implicated in the melanoma cell response to targeted therapy. Some studies have shown that selective inhibition of BRAF^V600E^ leads to increased MITF expression followed by upregulated PGC1α levels that facilitate drug resistance [17,27,69]. On the other hand, other studies, including our own, have shown that melanoma cells may lose MITF expression during acquisition of drug resistance [31,70,71]. In this context, both upregulation and downregulation of MITF expression may confer an advantage for melanoma cells treated with targeted therapeutics. Whether in this setting MITF downregulation in physiological normoxia provided direct advantage is unclear, however, it was concomitant with limited effects of drugs on the frequency of Ki-67-positive cells in normoxia (6% O_2_) when compared with drug effects in the atmospheric oxygen concentration (21%). Clinically, high proliferative index of recurrent melanomas, displayed as high Ki-67 expression, is an independent predictor of worse overall survival [72,73]. Our study indicates that the response of melanoma cells to treatment can be greatly altered by artificial levels of oxygen in cell culture. Downregulation of VEGF expression by vemurafenib and trametinib in hyperoxia falls into the same category; it is likely an artefact of 21% O_2_ in vitro culture and would presumably be disproved in a mouse model. It serves, however, as a proof of concept that an excessive oxygen concentration in cell culture may contribute data that cannot be reproduced in vivo. It is reasonable to assume that an opposite effect may also be observed where a tested compound is less effective in 21% O_2_ than it would be in physiologically relevant oxygen levels and becomes screened out before being investigated in vivo.

Concentration of oxygen plays an important role in maintenance of stemness, in normal tissue but also in cancer [74,75]. While in some studies, low oxygen levels promote stem like properties shown as increased NGFR level, in other studies low oxygen concentrations predominantly promote invasive phenotype with downregulated NGFR expression [76,77,78]. Both stem-like and invasive properties are connected with increased glycolysis and hypoxia [26,77,79]. All differences in the composition of subpopulations, namely NGFR-positive, MITF-positive and Ki-67-positive cells observed in this study between normoxia and hyperoxia, were cell-line specific but also point at oxygen as the causative factor of melanoma heterogeneity.

This is a preliminary study limited to a small sample number. Nevertheless, within just three patient-derived cell lines we observed distinct responses to normoxia, which demonstrate the diversity and plasticity of melanoma cells. A large scale comparison of metabolic phenotypes in varying oxygen concentrations would be required. We have shown that patient-derived melanoma cells in normoxia is a valid research model and that although additional adjustments in cell culture have to be made, melanoma cells can be successfully propagated in physiologically relevant oxygen levels in a regular manner. The application of normoxia research model could potentially reach beyond in vitro experiments as well. A comparative study of patient-derived xenografts (PDX) and cell line-derived xenografts (CDX) found transcriptome-wide differences between the two xenograft models that were attributed to divergent regulation of hypoxic response. Bhadury and colleagues concluded that cell lines became ‘pseudo-hypoxic’ when injected into mice, owing it to prior adaptation of these cells to standard laboratory culture conditions [80]. Arguably, tumor cells pre-adapted in vitro to an oxygen concentration relevant to in vivo settings might also prove advantageous in animal research models. Thus, this study, although preliminary, produced encouraging data that validates normoxia (6% O_2_) as a research model in vitro.

## 4. Materials and Methods

### 4.1. Tumor Tissues and Cell Culture

Patient-derived melanoma cells were obtained during surgical interventions [31,81]. The study was approved by Ethical Commission of Medical University of Lodz (identification code: RNN/84/09/KE). Each patient signed an informed consent before tissue acquisition. Tumor tissues were processed immediately after surgical procurement and suspensions of melanoma cells for culturing were generated within 2 h. Cells were named DMBC12, DMBC17, and DMBC28, (for Department of Molecular Biology of Cancer, DMBC). Melanoma cells were maintained in stem cell medium (SCM), consisting of DMEM/F12 medium, B-27 supplement (Gibco, Paisley, UK), insulin (10 μg/mL), heparin (1 ng/mL), 10 ng/mL bFGF, 20 ng/mL EGF (BD Biosciences, San Jose, CA, USA) and antibiotics (100 IU/mL penicillin, 100 μg/mL streptomycin). For initial part of the study involving proliferation assays, optimization of cell density, preliminary investigation of oxygen-dependent changes in gene expression as well as all experiments in hypoxia, cells were exposed to indicated oxygen concentration for the length of the assay. For all further analysis on physiological normoxia, melanoma cells were maintained in 6% for at least 3 weeks prior to experiments and all throughout the study. For experiments, cells were left to grow overnight before being treated with trametinib at the concentrations of 30 nM or 50 nM and vemurafenib at 5 μM or 10 μM. Then, cells were collected for RNA isolation (after 24 h), cell lysates (after 26 h or 44 h), viability analysis (after 72 h) and immunophenotype analysis (after 24 h or 48 h).

### 4.2. A Time-Lapse Fluorescence Microscopy

For proliferation analysis, a time-lapse fluorescence microscope system IncuCyte ZOOM (Essen Bioscience, Ann Arbor, MI, USA) was used. The data were analyzed using the IncuCyte ZOOM software. Proliferation was determined as the image area occupied by melanoma cells. Changes in the confluence are presented as fold-change relative to time T_0_.

### 4.3. Flow Cytometry

Flow cytometric data were acquired with FACSVerse (BD Biosciences, San Jose, CA, USA), and analyzed using BD FACSuite. For viability assay, cells were stained with Annexin V-FLUOS Staining Kit (Roche, Mannheim, Germany) for 15 min. For immunophenotype analysis, cells were left to grow overnight before being treated with drugs for 24 h or 48 h. LIVE/DEAD Fixable Violet Dead Cell Stain Kit (Life Technologies, Eugene, OR, USA) was used to exclude dead cells from analysis. For intracellular staining, cells were fixed with 4% paraformaldehyde and permeabilized with 0.1% Triton X-100 in PBS for 20 min. Antibodies against Ki-67 (Alexa Fluor647-conjugated) and NGFR (PE-conjugated) were from BD Biosciences, and antibodies against MITF (Alexa Fluor488-conjugated) were from Abcam (Cambridge, UK). Appropriate isotype controls were included in each experiment.

### 4.4. RNA Isolation, cDNA Synthesis, and Quantitative RT-PCR (qRT-PCR)

Total RNA was isolated and purified using Total RNA Isolation kit with mini column system (A&A Biotechnology, Gdynia, Poland). Total RNA (1 μg) was transcribed into cDNA using 300 ng of random primers and SuperScript II Reverse Transcriptase (Invitrogen Life Technologies, Carlsbad, CA, USA). The evaluation of mRNA expression of selected genes was performed by quantitative real-time polymerase chain reaction using the Rotor-Gene 3000 Real-Time DNA analysis system (Corbett Research, Mortlake, Australia). Amplification was performed using KAPA SYBR FAST qPCR Kit Universal 2X qPCR Master Mix (Kapa Biosystems, Cape Town, South Africa), 200 nM of each primer and 25 ng cDNA template per reaction. Primer sequences were as follows: GLS forward 5′-TGC ATT CCT GTG GCA TGT AT-3′ and reverse 5′-TTG CCC ATC TTA TCC AGA GG-3′, GLUT1 forward 5′-CGG GCC AAG AGT GTG CTA AA-3′ and reverse 5′-TGA CGA TAC CGG AGC CAA TG-3′, HK2 forward 5′-TAG GGC TTG AGA GCA CCT GT-3′ and reverse 5′-CCA CAC CCA CTG TCA CTT TG-3′, MITF-M forward 5′-ACC GTC TCT CAC TGG ATT GG-3′ and reverse 5′-TAC TTG GTG GGG TTT TCG AG-3′, PDK1 forward 5′-CTA TGA AAA TGC TAG GCG TCT-3′ and reverse 5′-AAC CAC TTG TAT TGG CTG TCC-3′, PGC1α forward 5′-AAC AGC AGC AGA GAC AAA TGC ACC-3′ and reverse 5′-TGC AGT TCC AGA GAG TTC CAC ACT-3′, RPS17 forward 5′-AAT CTC CTG ATC CAA GGC TG-3′ and reverse 5′-CAA GAT AGC AGG TTA TGT CAC G-3′, SLC1A5 forward 5′-GAG CTG CTT ATC CGC TTC TTC-3′ and reverse 5′-GGG GCG TAC CAC ATG ATC C-3′, SLC7A11 forward 5′-TGC TGG GCT GAT TTA TCT TCG-3′ and reverse 5′-GAA AGG GCA ACC ATG AAG AGG-3′, VEGF forward 5′-TTG CCT TGC TGC TCT ACC TCC A-3′ and reverse 5′-GAT GGC AGT AGC TGC GCT GAT A-3′. To calculate the relative expression of target genes vs. a reference gene RPS17, a mathematical model including an efficiency correction was used.

### 4.5. Western Blot

Melanoma cells were lysed in RIPA buffer containing 50 mmol/l Tris-HCl pH 8.0, 150 mmol/l NaCl, 1% Triton X-100, 0.5% sodium deoxycholate, 0.1% SDS supplemented with freshly added protease and phosphatase inhibitors (Merck, Darmstadt, Germany). Protein concentration was determined by Bradford assay. Cell lysates were diluted in 2 × Laemmli buffer (125 mM Tris-HCl pH 6.8, 0.004% bromophenol blue, 20% glycerol, 4% SDS and 10% β-mercaptoethanol) and protein samples were loaded on 7% SDS-polyacrylamide gel followed by electrophoresis at constant voltage 25 V/cm. The proteins were transferred onto an Immobilon-P PVDF membrane (Millipore, Billerica, MA, USA). The membrane was incubated in a blocking solution: 5% nonfat milk in PBS-Tween 0.05% or 5% phospho- BLOCKER (Cell Biolabs, San Diego, CA, USA) in PBS-Tween 0.05% for 45 min. Primary antibodies detecting ERK1/2, phospho-ERK1/2 (Thr^202^/Tyr^204^), AKT, phospho-AKT (Ser^473^), HIF-2α, MITF, PGC1α, MCL-1 and PARP were from Cell Signaling Technology (Danvers, MA, USA), PDH and GAPDH from Santa Cruz Biotechnology (Santa Cruz, CA, USA), phospho-PDH (Ser^293^) and β-actin from Merck (Darmstadt, Germany), and HIF-1α from BD Biosciences. Secondary HRP-conjugated anti-mouse antibodies were from Santa Cruz Biotechnology and anti-rabbit antibodies were from Cell Signaling. The membrane was incubated with Pierce^®^ ECL Western Blotting Substrate (Pierce, Rockford, IL, USA) for 1 min and the proteins were visualized using ChemiDoc Imaging System (BioRad, Hercules, CA, USA).

### 4.6. Protein Decay Assay

Melanoma cells were incubated with 100 µg/mL cycloheximide (Sigma-Aldrich, Saint Louis, MO, USA) to inhibit de novo protein synthesis. Cells were collected at 1 h, 2 h, 3 h, 4 h, 6 h and 10 h intervals after treatment with cycloheximide for cell lysate preparation and Western blotting.

### 4.7. Drugs

Vemurafenib and trametinib were purchased from Selleck Chemicals LLC (Houston, TX, USA).

### 4.8. Statistical Analysis

Graphs are presented as mean ± SD. Student’s *t*-test was performed to determine significant differences between the mean values of the tested parameters. The difference was considered significant if *p* < 0.05.

## Supplementary Materials

Supplementary Materials can be found at https://www.mdpi.com/1422-0067/20/17/4203/s1.

## Abbreviations

AKT	Protein Kinase B
AMPK	AMP-Activated Protein Kinase
AXL	Tyrosine-protein kinase receptor
bFGF	Basic fibroblast growth factor
CDX	Cell line-derived xenografts
CHX	Cycloheximide
DMBC	Department of Molecular Biology of Cancer
EGF	Epidermal growth factor
GAPDH	Glyceraldehyde 3-phosphate dehydrogenase
GLS	Glutaminase
GLUT1	Glucose Transporter Type 1
HIF-1α	Hypoxia Inducible Factor 1 Subunit Alpha
HIF-2α	Hypoxia Inducible Factor 2 Subunit Alpha
HK2	Hexokinase 2
MAPK	Mitogen-Activated Protein Kinase
MCL-1	Myeloid Cell Leukemia 1
MITF	Microphthalmia-Associated Transcription Factor
mTORC2	Mammalian target of rapamycin complex 2
NGFR	Nerve Growth Factor Receptor
OXPHOS	Oxidative phosphorylation
PDH	Pyruvate Dehydrogenase
PDK1	Pyruvate Dehydrogenase Kinase 1
PDX	Patient-derived xenografts
PGC1α	Peroxisome Proliferator-Activated Receptor Gamma Coactivator 1-Alpha
PI3K	Phosphatidylinositol-4,5-Bisphosphate 3-Kinase
RPS17	Ribosomal Protein S17
SLC1A5	Solute Carrier Family 1 Member 5
SLC7A11	Solute Carrier Family 7 Member 11
SOX10	Sex Determining Region Y-Box 10
TCA	tricarboxylic acid cycle
TME	Tumor microenvironment
TRA	Trametinib
VEGF	Vascular Endothelial Growth Factor
VEM	Vemurafenib
