# Supplementary material for: Physiologically Relevant Oxygen Concentration (6% O2) as an Important Component of the Microenvironment Impacting Melanoma Phenotype and Melanoma Response to Targeted Therapeutics In Vitro
Source: Int J Mol Sci. 2019 Aug 27;20(17):4203. doi: 10.3390/ijms20174203 (PMC6747123; doi:10.3390/ijms20174203)
Supplement: Supplementary file 1 [file ijms-20-04203-s001.pdf]

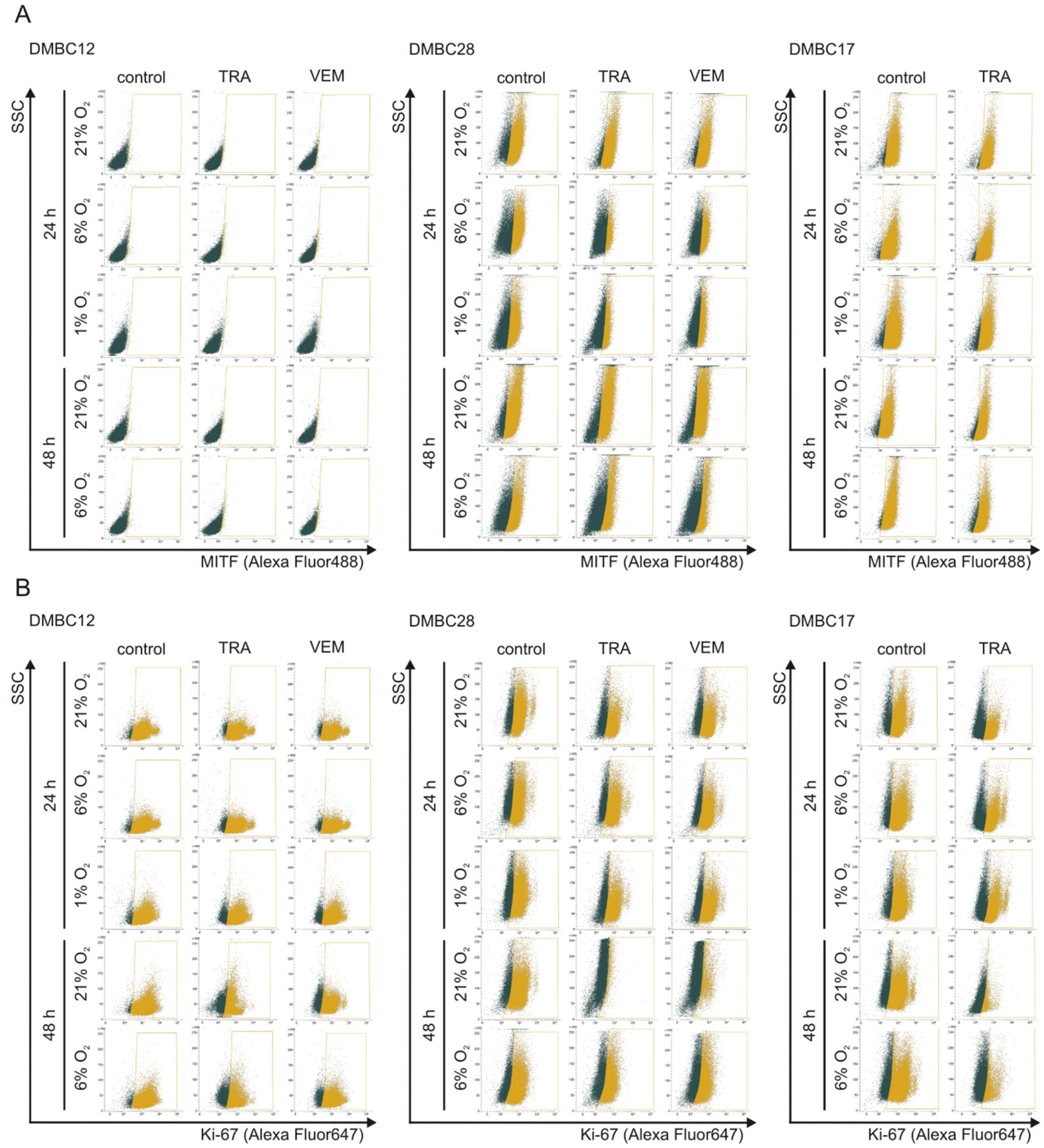

Supplementary Figure S1. Targeted therapeutics vemurafenib (VEM) and trametinib (TRA) reduce the frequency of Ki-67- and MITF-positive melanoma cells in a cell line- and oxygen-dependent manner. Representative dot plots of melanoma cells stained with anti-MITF (A) and anti-Ki-67 (B) antibodies after 24 h and 48 h of treatment with either 50nM TRA or 10μM VEM. Cells positive for each marker are labeled in yellow.
